# Threonine Phosphorylation and the Yin and Yang of STAT1: Phosphorylation-Dependent Spectrum of STAT1 Functionality in Inflammatory Contexts

**DOI:** 10.3390/cells13181531

**Published:** 2024-09-12

**Authors:** Maha M. Elbrashy, Hozaifa Metwally, Shuhei Sakakibara, Tadamitsu Kishimoto

**Affiliations:** 1 Laboratory of Immune Regulation, Immunology Frontier Research Center, The World Premier International Research Center Initiative (WPI), Osaka University, Osaka 565-0871, Japan; mahahosny36@ifrec.osaka-u.ac.jp (M.M.E.); sakakibara@ifrec.osaka-u.ac.jp (S.S.); 2 Biochemistry Department, Biotechnology Research Institute, National Research Center, Giza P.O. Box 12622, Egypt; 3 Graduate School of Medical Safety Management, Jikei University of Health Care Sciences, Osaka 532-0003, Japan

**Keywords:** threonine, phosphorylation, STAT1, interferons, lupus, inflammation, autoimmunity

## Abstract

Threonine phosphorylation promotes inflammatory functions of STAT1 while restricting its interferon (IFN) signaling in innate immune responses. However, it remains unclear whether the restriction of STAT1-mediated IFN signaling conferred by threonine phosphorylation is a ubiquitous mechanism or one that is context-dependent. To address this, we utilized pristane-induced lupus, a prototype IFN-driven systemic autoimmune disease model characterized by the production of high-titer autoantibodies against nucleic acid-associated antigens. Through genetic and biochemical assays, we demonstrate that Thr748 phosphorylation is dispensable for STAT1 functionality in pristane-induced lupus. Genetically engineered mice expressing the phospho-deficient threonine 748-to-alanine (T748A) mutant STAT1 exhibited similar survival rates, high titers of anti-dsDNA IgG, and nephritis compared to their wild-type littermates. In sharp contrast, STAT1 deficiency protected mice against pristane-induced lupus, as evidenced by increased survival, low titers of anti-dsDNA IgG, and less severe nephritis in the STAT1 knockout mice compared to their T748A littermates. Our study suggests a phosphorylation-dependent modularity that governs the spectrum of STAT1 functionality in inflammatory contexts: IFN phospho-tyrosine-dependent and inflammatory phospho-threonine-dependent, with Thr748 phosphorylation driving selective inflammatory activities, particularly those not driven by the canonical JAK pathway. From a broader perspective, our findings provide deeper insights into how distinct phosphorylation events shape the combinatorial logic of signaling cassettes, thereby regulating context-dependent responses.

## 1. Introduction

Protein phosphorylation is a powerful tool utilized by signaling cassettes to rapidly mount and modify cellular responses to diverse environmental stimuli. Phosphorylation increases an organism’s proteome complexity far beyond what is conferred by the genome alone, enabling cells to recognize and respond to various environmental stimuli using a limited number of signaling cassettes [1,2,3]. This is best illustrated by the Janus kinase (JAK)–signal transducers and activators of transcription (STATs) pathway, which has represented a paradigm for membrane-to-nucleus signaling since its discovery over three decades ago from experiments on the antiviral mechanisms of interferons (IFNs) [4]. The canonical JAK–STAT signaling posits that STAT proteins remain latent in the cytoplasm until phosphorylated on a key tyrosine residue within their C-terminal transactivation domain (TAD), which is mediated by a receptor-associated JAK complex [5]. Despite having only four JAKs (JAK1, JAK2, JAK3, and TYK2) and seven STATs (STAT1, STAT2, STAT3, STAT4, STAT5A, STAT5B, and STAT6), this pathway can process signals from a vast array of ligands, including cytokines, growth factors, and hormones [4,6]. Additionally, emerging evidence has shown that STAT proteins contribute JAK-independent activities in maintaining cellular homeostasis and responding to various environmental stimuli, expanding the traditional view of their functionality and regulatory mechanisms beyond the canonical JAK pathway [7,8]. This versatility is achieved through the combinatorial use of different JAK and STAT proteins, allowing for specific and context-dependent cellular responses in various physiological processes, such as immune response, cell growth, and differentiation.

Stat1 is ubiquitously expressed and is comprised of a full-length α isoform and its splice variant β isoform, which lacks the last 38 amino acids of its TAD, suggesting a possible contribution of these amino acids to the specificity of the transcriptional activities of Stat1 [4]. The JAK-mediated Tyr701 phosphorylation of Stat1 is central for all types of IFN signaling and the transcription of IFN-stimulated genes (ISGs) [9]. Several post-translational modifications within the TAD contribute to the selectivity of the transcriptional activity of Stat1 following IFN stimulation [10,11,12,13]. In addition to its ubiquitous IFN signaling, STAT1 can also alter inflammation in a context-specific manner, yet the scope and mechanisms remain incompletely characterized. Our research has uncovered that Thr748 phosphorylation of STAT1 drives inflammatory responses while restricting IFN signaling in innate immune responses following lipopolysaccharide (LPS) stimulation [14,15]. The identification of Thr748 phosphorylation opens the door for exploring the combinatorial signaling logic within a single STAT protein, where different modifications, such as phosphorylation, modulate the protein’s output functions. This adds another layer of signal integration and regulation, achieving complex cell-specific and context-dependent responses.

Systemic lupus erythematosus (SLE) is a systemic autoimmune disease that predominantly affects females and is characterized by a strong “IFN signature” in patients’ peripheral blood and tissues such as the skin and kidneys [16]. Additionally, recombinant IFN induces lupus-like symptoms like those observed in spontaneous SLE [17]. Furthermore, genome-wide association studies highlight the contribution of type I IFN signaling to SLE susceptibility [18]. In this study, we investigate whether the Thr748 phosphorylation-mediated restriction of IFN-STAT1 signaling is a ubiquitous mechanism or context-dependent. Using a murine model for SLE, a prototype “type I interferonopathy,” we demonstrate that Thr748 phosphorylation contributes selective and context-dependent STAT1 activities, particularly those not driven by the canonical JAK pathway.

## 2. Materials and Methods

### 2.1. Mice

Stat1T748A mutant and Stat1KO mice on a C57BL/6 background were generated as previously described [15]. Mice were kept and bred in pathogen-free conditions. Age-matched female littermates were used for the experiments. All animal experiments were conducted in accordance with the guidelines of the Animal Care and Use Committee of Osaka University.

### 2.2. Pristane-Induced Lupus

Seven- to eight-week-old female littermate mice were injected intra-peritoneally with 0.5 mL pristane (P2870 Sigma, Darmstadt, Germany) and followed up over for 28 weeks. Mice were analyzed for survival, serum titers of anti-dsDNA, and proteinuria.

### 2.3. Enzyme-Linked Immunosorbent Assay (ELISA)

Serum titers of anti-dsDNA were measured by LBIS Mouse anti-dsDNA ELISA Kit (FUJIFILM Wako, Neuss, Germany). Albuminuria was measured as μg albumin per mg creatinine from midstream urine using Mouse Urinary Albumin Quantitative ELISA Kit (Ethos Bioscience, Logan Township, NJ, USA) and Urinary Creatinine Quantitation Kit Creatinine Companion (Ethos Bioscience, Logan Township, NJ, USA) according to the manufacturer’s protocol.

### 2.4. Histopathological Staining and Analysis

For assessing glomerulonephritis, kidneys collected from naïve or pristane-treated mice were fixed with 4% PBS-buffered paraformaldehyde for 1 h. Then, the buffer was changed to 10% followed by 20% PBS-buffered saccharide. Histopathological specimen preparation and hematoxylin–eosin (H&E) staining were prepared by the Kyoto Institute for Nutrition and Pathology, Kyoto, Japan. Images were taken using a BZ-X710 microscope (KEYENCE, Osaka, Japan).

### 2.5. Immunofluorescent Staining and Analysis

For assessing immune complex depositions in glomeruli, kidneys collected from naïve or pristane-treated mice were fixed in PBS containing 4% paraformaldehyde and then incubated in 15% sucrose in PBS overnight. Kidneys were embedded in OCT compound (Sakura Finetek, Tokyo, Japan) and kept at −80 °C. Frozen tissues were sliced, then placed on a glass slide, and stained with hematoxylin and eosin (Kyoto Institute of Nutrition and Pathology, Inc., Kyoto, Japan). For fluorescent staining, unstained slides were washed with PBS, followed by blocking with PBS containing 0.1% Triton, 1% bovine serum albumin (BSA), and 5% fetal bovine serum (FBS), for 15 min at room temperature. The sections were incubated with the same buffer containing goat anti-mouse IgG conjugated with Alexa Fluor 488 (Cat: A11001, Thermo, Waltham, MA, USA). After washing with PBST, the slide was mounted with a Vectashield mounting medium containing DAPI (Vector Laboratories, Newark, CA, USA). Images were acquired by the laser scanning confocal microscope (FV1000, Olympus, Shinjuku, Tokyo, Japan) equipped with ×60 objective lens. Image files were converted into PNG files using the Fluoview viewer software version 10 (Olympus, Shinjuku, Tokyo, Japan).

### 2.6. Preparation of Single Cell Suspension of Splenocytes

Mice were euthanized using CO_2_ overdose and immediately dissected. The spleen was collected, gently mashed, and suspended in 10 mL of RBC lysis buffer. After lysis of the red blood cells, the remaining cells were washed with cell isolation buffer, which consists of ice-cold PBS with 10% FBS (*v*/*v*). The cell suspension was then centrifuged at 300× *g* for 5–10 min at 4 °C. The cell pellet was washed again with cell isolation buffer, passed through a 70 μm cell strainer into a 50 mL conical tube, and centrifuged once more at 300× *g* for 5–10 min at 4 °C. Finally, the pellet containing splenocytes was resuspended in an appropriate volume of RIPA lysis buffer (Thermo Fisher, Waltham, MA, USA) for protein analysis or TRIzol Reagent (Thermo Fisher, Waltham, MA, USA) for RNA analysis.

### 2.7. RT-qPCR

Cells were lysed in 0.5 mL of TRIzol Reagent (Thermo Fisher, Waltham, MA, USA), and total RNA was extracted using Direct-zol RNA MicroPrep (Zymo Research, Tustin, CA, USA). Then, cDNA was prepared using SMART MMLV RT (Takara, Kusatsu, Japan), Advantage UltraPure dNTP (Takara, Kusatsu, Japan), and Oligo d(T)23 VN (New England Biolabs, Ipswich, MA, USA) according to the manufacturer’s instructions. Real-time qPCR was performed on the generated cDNA (5–10 ng/rxn) using pre-designed TaqMan primers (Thermo Fisher, Waltham, MA, USA) listed in (Table 1). The RT-qPCR was performed using TaqMan Fast Advanced Master Mix (Thermo Fisher, Waltham, MA, USA) in a 96-well plate using QuantStudio 3 Real-Time PCR System (Thermo Fisher, Waltham, MA, USA). Relative expression was calculated from the Ct values.

### 2.8. Immunoblotting

Whole-cell lysates were prepared using RIPA Lysis and Extraction Buffer (Thermo Fisher, Waltham, MA, USA). Protein concentration was quantified using Pierce Detergent Compatible Bradford Assay Kit (Thermo Fisher, Waltham, MA, USA). Lysates were boiled in SDS sample buffer (Nacalai Tesque, Kyoto, Japan) for 5 min at 96 °C; 10 to 20 µg of total protein was loaded and resolved by 5–20% SDS-PAGE gels (Nacalai Tesque, Kyoto, Japan). Proteins were then transferred onto 0.45 µM polyvinylidene difluoride (PVDF) membrane (GE Healthcare, Chicago, IL, USA) by wet transfer system using Criterion Blotter (BioRad, Hercules, CA, USA), washed, and incubated with primary antibodies. Then, the membrane was washed thrice and incubated with anti-rabbit IgG horseradish peroxidase (HRP) conjugate, blots were developed, and images were taken by an Image Quant LAS800 (GE Healthcare, Chicago, IL, USA). Antibodies used are listed in Table 2.

### 2.9. Statistical Analysis and Illustrations

Survival was analyzed using Kaplan–Meier curves and log-rank (Mantel–Cox) test. One-way ANOVA with post hoc Tukey’s test and unpaired two-tailed Student’s *t*-test with Welch’s correction were used to determine *p* values. *p* value < 0.05 was considered statistically significant. All statistical analyses were performed using GraphPad Prism software version 9. Immunoblot band intensity was measured by ImageJ (v1.54g). Figures were prepared by Adobe Illustrator (v28.6 (2024)), and schematic diagrams were drawn using BioRender.

## 3. Results

### 3.1. T748A Mice Exhibited Similar Survival Rate and High Titers of Autoantibodies Compared to Their Wt Littermates Following Pristane-Induced Lupus

Intraperitoneal injection of pristane, a mineral oil, induces peritoneal irritation in mice, leading to the development of a lupus-like disease after several months [19,20]. This disease is characterized by the production of autoantibodies against nucleic acids [21]. Similar to over half of lupus patients, pristane-induced lupus in mice is highly dependent on the overproduction of type I interferons IFNs [22,23,24]. In C57BL/6 mice, following pristane injection, approximately 50% succumb to pulmonary hemorrhage, resembling diffuse alveolar hemorrhage (DAH), a devastating complication for lupus patients [25,26,27].

**Figure 1:**
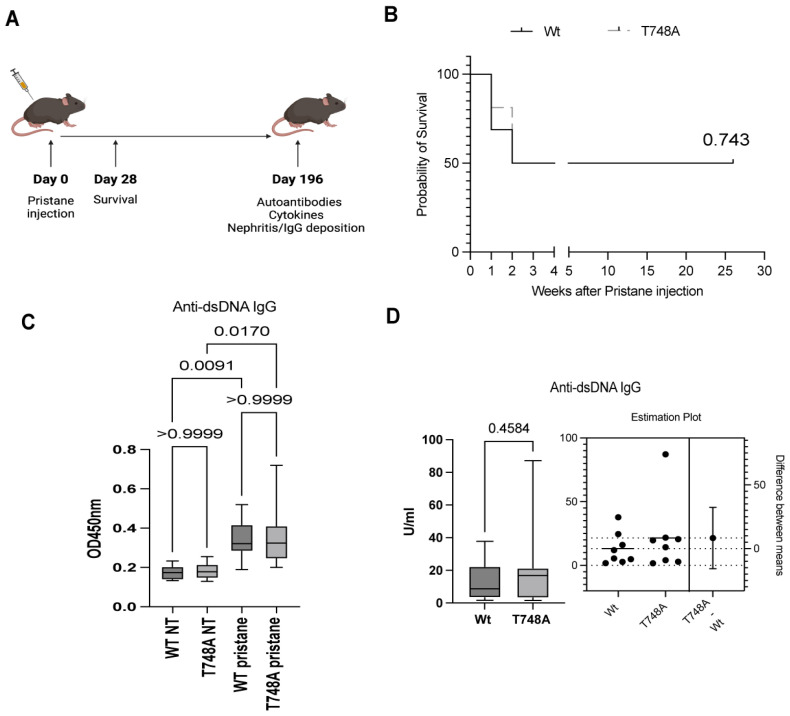
T748A mice exhibited similar survival rates and high titers of autoantibodies compared to their Wt littermates following pristane-induced lupus. (**A**) Schematic diagram of experimental design; (**B**) survival rate of pristane-injected Wt and T748A littermates (*n* = 16 mice per genotype); (**C**,**D**) serum levels of anti-dsDNA IgG of naïve and pristane-injected Wt and T748A littermates as measured by ELISA (*n* = 8 mice per genotype per group). *p* values are shown as measured by log-rank test (Mantel-Cox) (**B**), one-way ANOVA with post hoc Tukey’s test (**C**), or unpaired student’s *t* test with Welch’s correction (**D**). NT, non-treated.

To investigate the role of the Thr748 phosphorylation of STAT1 in the lupus pathology, we examined the survival rates, titers of autoantibodies, cytokines expression, and immune complex glomerulonephritis in wild type (hereafter referred to as Wt) and their Stat1T748A mutant (hereafter referred to as T748A) female littermates on C57BL/6 background following pristane intraperitoneal injection (Figure 1A). As expected, 50% of Wt mice (8 out of 16 survived) succumbed within 4 weeks following pristane injection (Figure 1B). Intriguingly, similar to their Wt littermates, pristane-injected T748A mice exhibited 50% survival (8 out of 16 survived) (Figure 1B). We next measured the titers of anti-dsDNA IgG in the serum of naïve and following 28 weeks after pristane injection of Wt and T748A littermates. Indeed, both Wt and T748A littermates produced high titers of anti-dsDNA IgG following pristane injection (Figure 1C). However, the anti-dsDNA IgG titers were comparable between pristane-injected Wt and T748A littermates (Figure 1C,D). Our observations show that the Thr748 phosphorylation of STAT1 is dispensable for survival and autoantibody production in pristane-induced lupus.

### 3.2. Thr748 Phosphorylation Is Dispensable for Stat1-Mediated Cytokines Expression and IFN Signaling Following Pristane-Induced Lupus

Cytokines are pivotal in the pathogenesis of lupus, driving the immune responses that cause inflammation and tissue damage [28]. IL-6 facilitates the differentiation of B cells into plasma cells that produce autoantibodies, with elevated IL-6 levels correlating with disease activity and severity, particularly in lupus nephritis [29]. IL-12B promotes the differentiation of naive T cells into Th1 cells, which secrete interferon-gamma (IFN-γ), fostering an inflammatory environment and activating autoreactive T cells [30]. TNF promotes inflammation and can induce cell apoptosis, contributing to tissue damage and the continuation of the autoimmune response; higher TNF levels are associated with increased disease activity and organ involvement [31]. IL-10, typically an anti-inflammatory cytokine, paradoxically enhances B-cell survival and antibody production in lupus patients, worsening the disease despite its usual role in limiting immune responses [32]. Type I interferons are central for lupus pathogenesis, activating dendritic cells, B cells, and T cells, and boosting autoantibody production [33].

**Figure 2:**
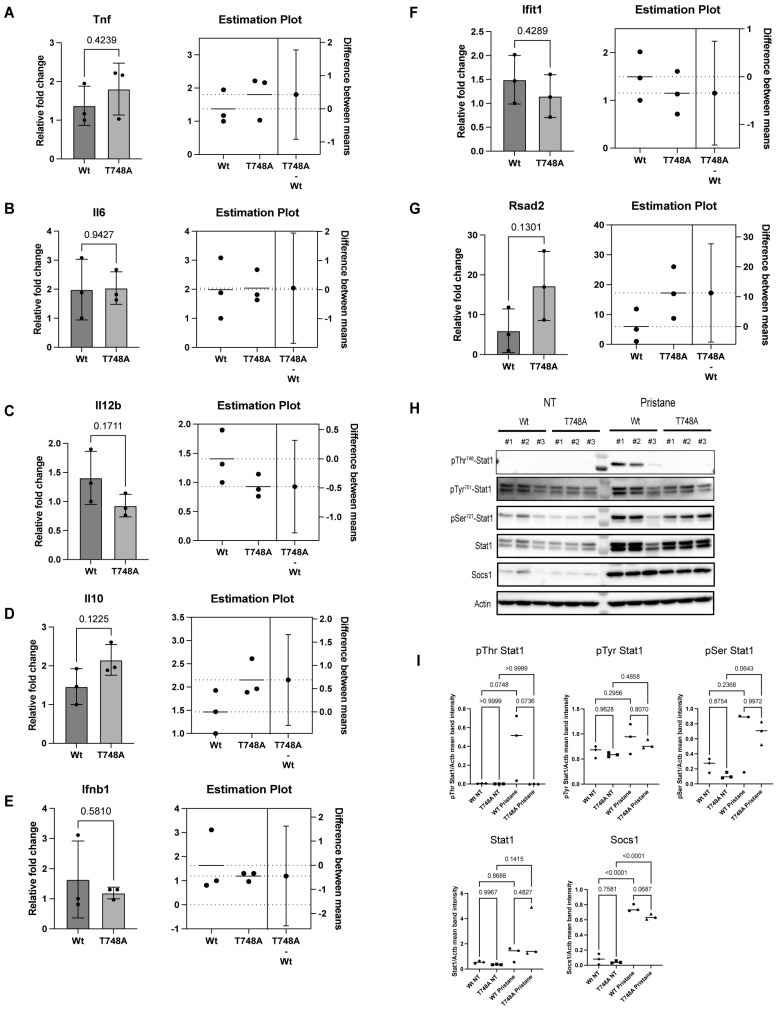
Thr748 phosphorylation is dispensable for Stat1-mediated cytokines expression and IFN signaling following pristane-induced lupus. (**A**–**G**) Splenocytes from pristane-injected Wt and T748A littermates. Total RNA was isolated, and the indicated transcripts were quantified by qRT PCR. Data are presented as medians (*n* = 3 biological replicates). (**H**) Splenocytes from naïve and pristane-injected Wt and T748A littermates. Whole-cell lysates were harvested and separated by SDS PAGE. The indicated endogenous proteins were detected by Western blotting analysis. Data show three independent biological replicates per genotype per group. (**I**) Quantification of mean band intensities of (**H**). *p* values are shown as measured by unpaired student’s *t* test with Welch’s correction (**A**–**G**) and one-way ANOVA with post hoc Tukey’s test (**I**). NT, non-treated.

The spleen provides a representative sample of the systemic immune response, reflecting the activation status of immune cells, cytokine production, and the presence of autoreactive cells [34]. In SLE, the dysregulation of these immune cells leads to autoantibody production, immune complex formation, and chronic inflammation, which are key contributors to the disease’s characteristic symptoms and organ damage [35]. Therefore, analyzing splenocytes is valuable for monitoring SLE disease activity. We, therefore, examined whether the Thr748 phosphorylation of STAT1 contributes to cytokines expression following pristane injection by analyzing their expression levels in splenocytes. Notably, T748A mice exhibited similar levels of expression of inflammatory cytokines such as Tnf, Il6, Il12b, and Il10 compared to their Wt littermates (Figure 2A–D). Additionally, levels of expression of Ifnb and ISGs such as Ifit1 and Rsad2 were comparable between T748A and Wt littermates (Figure 2E–G). We then investigated whether the lack of requirement for Thr748 phosphorylation of STAT1 in the observed lupus pathology is due to its dispensability for STAT1 function in the pristane-induced lupus model, or whether pristane fails to induce Thr748 phosphorylation in Wt mice as well. Remarkably, pristane induced Thr748 phosphorylation of Stat1 in Wt, unlike their T748A littermates (Figure 2H,I). In contrast, pristane induced comparable IFN-mediated Stat1 signaling between Wt and T748A littermates as shown by the expression of the canonical phospho-Tyr701 and phospho-Ser727 following pristane injection (Figure 2H,I). Furthermore, the expression of the negative regulator of IFN-Stat1 signaling, Socs1, was comparable between pristane-injected Wt and T748A littermates (Figure 2H,I). Thus, our ex vivo analysis indicates that the Thr748 phosphorylation is dispensable for Stat1-mediated lupus pathology following pristane injection.

### 3.3. T748A Mice Exhibited Similar Albuminuria and Glomerulonephritis Compared to Their Wt Littermates Following Pristane-Induced Lupus

Lupus nephritis (LN) is one of the most severe complications of SLE, associated with immune complex deposition in the glomeruli, which triggers an inflammatory response that damages kidney tissues [36]. This inflammatory process is driven by autoantibodies and the subsequent activation of the complement system, resulting in glomerulonephritis [37]. Albuminuria, the presence of albumin in the urine, is a key clinical marker of LN, indicating the inflammation and damage to the glomeruli that increase their permeability, allowing albumin to leak into the urine [38]. At the histopathological level, LN is evaluated by examining several key pathological features comprising glomeruli involvement, tubulointerstitial changes, and vascular involvement. The extent of glomerular involvement is assessed through mesangial hypercellularity, endocapillary proliferation, and crescent formation [39]. Tubulointerstitial changes include tubular atrophy, interstitial inflammation, and fibrosis [40]. Vascular involvement includes vasculitis, thrombotic microangiopathy, and immune complex deposition within vessel walls, and vascular lesions often indicate more severe disease and a higher risk of chronic kidney disease [41]. Besides this, common immunofluorescence findings include deposits of IgG in subendothelial, subepithelial, and mesangial locations, which are indicative of LN [42].

**Figure 3:**
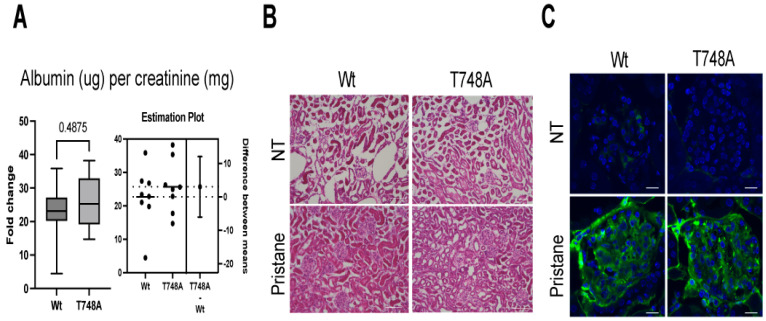
T748A mice exhibited similar albuminuria and glomerulonephritis compared to their Wt littermates following pristane-induced lupus. (**A**) Measurements of the levels of albumin/creatinine in the urine of pristane-injected Wt and T748A littermates as measured by ELISA. (**B**) Histopathological analysis of glomerulonephritis. Scale bar, 100 μm. (**C**) Immunofluorescent analysis of autoantibodies deposition in glomeruli. Scale bar, 50 μm. Data are representative of two independent experiments. *n* = 8 mice per genotype per group for each experiment. *p* values are shown as measured by unpaired student’s *t* test with Welch’s correction (**A**). NT, non-treated.

By measuring LN, we examined whether the Thr748 phosphorylation of STAT1 contributes to the severity and progression of pristane-induced lupus. We first examined albuminuria in pristane-injected T748A or Wt littermates. Of note, T748A and Wt littermates exhibited similar levels of albuminuria as measured by the albumin/creatinine ratio (Figure 3A). In consistence with their albuminuria, pristane-injected T748A and Wt littermates exhibited similar levels of mesangial hypercellularity, endocapillary proliferation, crescent formation, interstitial inflammation, and vascular changes as shown by the histopathology analysis of their kidneys at naïve and following pristane injection conditions (Figure 3B). Furthermore, immunofluorescence analysis showed comparable IgG autoantibodies deposition in subendothelial, subepithelial, and mesangial locations of kidney glomeruli of pristane-injected T748A or Wt littermates (Figure 3C). These findings demonstrate that Thr748 phosphorylation of STAT1 is dispensable for the progression of pristane-induced lupus and the development and severity of LN.

### 3.4. Thr748 Phosphorylation Is Dispensable for STAT1-Mediated Pathology in Pristane-Induced Lupus

The mouse strain background significantly influences the development and severity of pristane-induced lupus [19]. BALB/c and C57BL/6 are commonly used strains with distinct responses to pristane injection, which induces lupus-like disease characterized by the production of autoantibodies and immune complex glomerulonephritis [43]. In BALB/c mice, pristane injection leads to a robust lupus-like disease, with high titers of autoantibodies against nucleic acids and severe immune complex glomerulonephritis [44,45]. On the other hand, C57BL/6 mice develop low-grade autoimmunity with lower titers of autoantibodies and less severe glomerulonephritis but also develop pulmonary hemorrhage, resembling DAH, a severe complication seen in some lupus patients [26,27,43,46].

**Figure 4:**
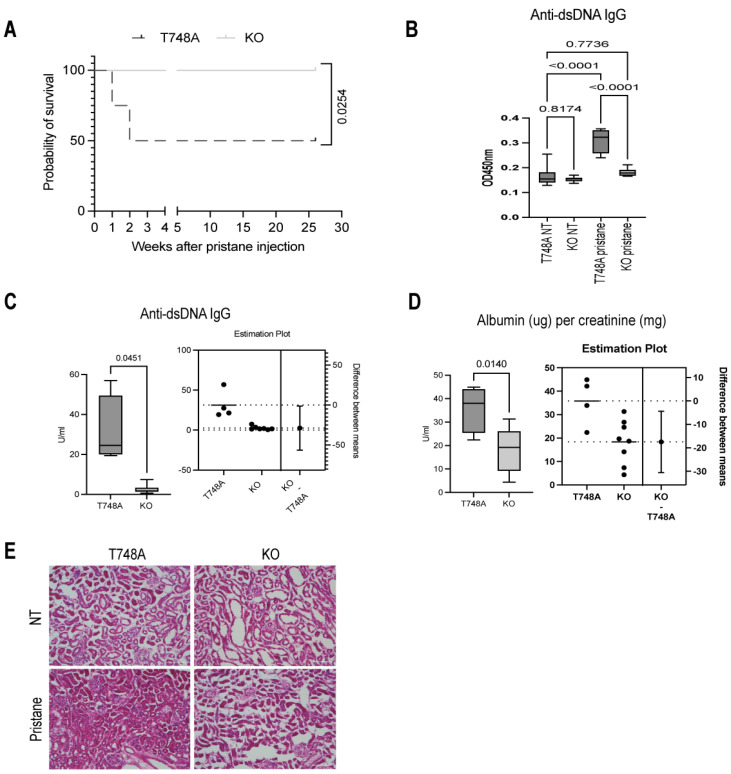
Thr748 phosphorylation is dispensable for STAT1-mediated pathology in pristane-induced lupus. (**A**) Survival rate of pristane-injected KO and T748A littermates. (**B**,**C**) Serum levels of anti-dsDNA IgG of naïve and pristane-injected KO and T748A littermates as measured by ELISA. (**D**) Measurements of the levels of albumin/creatinine in urine of pristane-injected KO and T748A littermates as measured by ELISA. (**E**) Histopathological analysis of glomerulonephritis. Data are representative of two independent experiments. *n* = 4–8 mice per genotype per group for each experiment. Scale bar, 100 μm. *p* values are shown as measured by log-rank test (Mantel-Cox) (**A**), one-way ANOVA with post hoc Tukey’s test (**B**), or unpaired student’s *t* test with Welch’s correction (**C**,**D**). NT, non-treated.

We, therefore, investigated whether the dispensability of the Thr748 phosphorylation in pristane-induced lupus is due to its lack of necessity for STAT1 function in the context of this IFN-driven disease, or if it is attributed to the mouse strain and the overall lower disease severity and dependency on total STAT1. To address this point, we compared the lupus severity of T748A and Stat1 knockout (hereafter referred to as KO) female littermates following pristane injection. Remarkably, deficiency of Stat1 protected the mice against pristane-induced lethality as demonstrated by the 100% survival of KO mice (16 out of 16 survived) compared to the 50% survival of their T748A littermates (4 out of 8 survived) (Figure 4A). We next measured the titers of anti-dsDNA IgG in the serum of naïve and following 28 weeks after pristane injection of KO and T748A littermates. Compared to their T748A littermates, KO mice did not develop high titers of anti-dsDNA IgG following pristane injection (Figure 4B). Notably, the anti-dsDNA IgG titers were significantly higher in the serum of pristane-injected T748A mice compared to their KO littermates (Figure 4B,C). Moreover, pristane-injected T748A mice exhibited higher levels of albuminuria as measured by the albumin/creatinine ratio compared to their KO littermates (Figure 4D). In consistence with their albuminuria, pristane-injected T748A mice exhibited severe mesangial hypercellularity, endocapillary proliferation, crescent formation, interstitial inflammation, and vascular changes compared to their KO littermates as shown by the histopathology analysis of their kidneys at naïve and following pristane injection conditions (Figure 4E). Our findings show that Stat1 drives the pathology of pristane-induced lupus independently of the Thr748 phosphorylation.

## 4. Discussion

Since its discovery over three decades ago, STAT signaling has garnered significant attention. The expanding body of knowledge on STAT signaling has not only deepened our understanding of cellular processes but has also been leveraged to develop biologics for treating various diseases [47]. Despite extensive research, a critical aspect remains elusive: Understanding how context-specific regulation of STAT signaling is achieved. A plausible explanation lies in combinatorial signaling logic [48]. However, the details of this combinatorial signaling logic and the modularity of STAT signaling remain largely unknown. We have identified Thr748 phosphorylation as an inflammatory controller of Stat1, which restricts its IFN signaling following LPS stimulation [15]. Threonine phosphorylation of Stat1 does not impact its nuclear translocation. Instead, it modulates Stat1’s DNA binding to non-canonical motifs, distinct from classical IFN-responsive elements, thereby activating the transcription of genes beyond the canonical ISGs [14]. The combinatorial signaling output of the tyrosine and threonine phosphorylation dictates the final output of Stat1 functionalities in shaping the innate immune response of macrophages towards pro- or anti-inflammatory responses, ultimately influencing host survival following the LPS challenge [15].

Using pristane-induced lupus, a prototype systemic interferonopathy, we show that Stat1 mediates lupus pathology independently of Thr748 phosphorylation. Complementary genetic and biochemical evidence supports this conclusion. Genetically, KO mice are resistant to pristane-induced lupus, while T748A mice exhibited similar pathology to their Wt littermates, as evidenced by lower survival rates, higher anti-dsDNA IgG titers, and lupus nephritis. Biochemically, the induction of Thr748 phosphorylation of STAT1 in pristane-injected Wt mice was dispensable for the observed pathology, as indicated by comparable cytokine expression and IFN-STAT1 signaling between Wt and T748A littermates. The simplest interpretation of these observations is that Thr748 phosphorylation is dispensable for STAT1-mediated pathology in pristane-induced lupus, indicating that Thr748 phosphorylation modulates Stat1 activities in a context-dependent manner. The context-dependency of Thr748 phosphorylation and its contribution to selective STAT1 functions in inflammatory contexts can be attributed, at least in part, to cell-specific signaling machinery. For instance, in macrophages, Thr748 phosphorylation of STAT1 is induced in a TLR4-MYD88-independent manner following LPS stimulation [14,15]. Conversely, lupus pathology is largely driven by IFN production through TLR7-MYD88-dependent signaling in B cells [49,50,51]. This is underscored by the fact that, over the past two decades, only two drugs have been approved by the FDA for treating SLE or LN: belimumab, a monoclonal antibody against the IFN-induced B-cell proliferation and differentiation factor BAFF, and anifrolumab, a monoclonal antibody blocking the type I IFN receptor [52,53]. Thus, distinct signaling machineries in macrophages and B cells may contribute to the context-dependent activation of Thr748 phosphorylation, thereby influencing the overall functions of STAT1 in different inflammatory contexts. Future research exploring the potential cell-specific spatiotemporal regulation of distinct phosphorylation downstream of the receptor will offer deeper insights into Stat1’s combinatorial logic and functionality across various biological contexts. While IFN-induced Tyr701 phosphorylation of Stat1 drives its homo- or heterodimerization with Stat2 and Irf9 [48], it would be intriguing to investigate whether Thr748 phosphorylation modulates Stat1’s interactions with other transcriptional co-factors in a cell-specific and context-dependent manner.

Extensive research on STAT1 has uncovered tyrosine and serine phosphorylation, along with other post-translational modifications, yet the potential functionality of STAT1 beyond IFNs and phospho-tyrosine signaling remains poorly understood. To fill this gap, the term unphosphorylated STAT1 (U-STAT1) was adopted to explain STAT1 functions beyond JAK phospho-tyrosine signaling. However, U-STAT1 expression itself relies on IFN-JAK signaling and enhances the expression of ISGs, leaving the long-standing question of how STAT1 contributes to JAK-independent activities unanswered [54,55,56]. Our recent findings uncover Thr748 phosphorylation as a potential mechanism for achieving cell-specific and context-dependent activities of STAT1, particularly those not driven by the JAK pathway [15]. Our observations mandate revisiting the current term U-STAT and open the door for exploring other potential functional threonine phosphorylation sites in the TAD of STAT proteins, which may provide a deeper understanding of STAT proteins’ functionalities beyond the canonical JAK signaling.

From a clinical perspective, while drugs targeting the JAK–STAT pathway are effective in treating various inflammatory and autoimmune diseases, they often carry significant adverse effects [57]. These therapies can disrupt the normal functioning of the immune system, leading to increased susceptibility to infections and malignancies [58]. Additionally, patients may experience hematologic abnormalities, such as anemia and thrombocytopenia, as well as gastrointestinal issues like diarrhea and nausea [59]. Targeting the JAK–STAT1 pathway can also interfere with lipid metabolism, resulting in hyperlipidemia and increasing the risk of cardiovascular events [60]. These adverse effects underscore the need for more selective therapeutic strategies that can mitigate inflammation without broadly compromising the immune system. A better understanding of Thr748 phosphorylation of STAT1 could unveil potentially pharmacologically targetable molecules and offer opportunities for developing more specific treatment modalities for inflammatory diseases. This approach could help avoid the adverse effects associated with targeting the JAK signaling pathway or the entire STAT1 protein.

## 5. Conclusions

**Figure 5:**
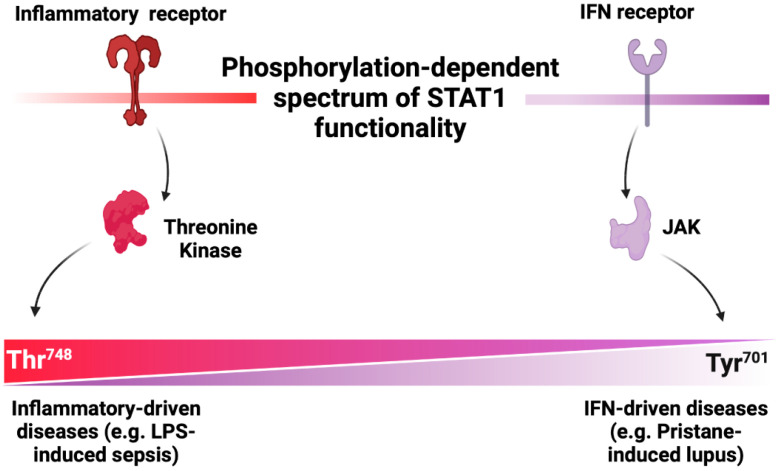
Scheme for a phosphorylation-dependent spectrum of STAT1 functionality in inflammatory contexts: IFN phospho-tyrosine-dependent and inflammatory phospho-threonine-dependent.

Our study offers strong proof-of-concept evidence for a phosphorylation-dependent spectrum of STAT1 functionality in inflammatory contexts, highlighting Thr748 phosphorylation as a driver of selective inflammatory activities, especially those independent of the canonical JAK pathway (Figure 5). Our findings shed new light on the combinatorial signaling logic of STAT1 in modulating context-dependent inflammatory responses, which could apply to other signaling pathways as well. Broadly, our observations enhance our understanding of how phosphorylation events influence signaling molecules’ capacity to elicit cell-specific and context-dependent responses to diverse stimuli.

## 6. Patents

H.M. and T.K. are inventors on the patent application (PCT/JP2023/022262) covering parts of the methodology in the presented work.

## Figures and Tables

**Table 1:** RT-qPCR primers list.

Gene	Manufacture	Catalog No.
*Gapdh*	TaqMan	Mm99999915_g1
*Il6*	TaqMan	Mm00446190_m1
*Il12b*	TaqMan	Mm01288989_m1
*Ifnb1*	TaqMan	Mm00439552_s1
*Tnf*	TaqMan	Mm00443258_m1
*Ifit1*	TaqMan	Mm00515153_m1
*Rsad2*	TaqMan	Mm00491265_m1
*Il10*	TaqMan	Mm01288386_m1

**Table 2:** Immunoblotting antibodies list.

Antibody	Manufacture	Catalog No.
Beta-actin (13E5) rabbit mAb	CST (Danvers, MA, USA)	4970S
P-Stat1 (Y701) (58D6) rabbit mAb	CST	9167L
P-Stat1 (S727) (D3B7) rabbit mAb	CST	8826S
Stat1 (D1K9Y) Rabbit mAb	CST	14994S
SOCS1 (E4K7Q) rabbit mAb	CST	68631S
P-Stat1 (Thr748) rabbit mAb	In-house	As previously described [15]
Anti-rabbit IgG, HRP-linked antibody	CST	7074S

## Data Availability

All data generated or analyzed during this study are included in this published article.
